# Sub‐Second Time‐Resolved Surface‐Enhanced Raman Spectroscopy Reveals Dynamic CO Intermediates during Electrochemical CO_2_ Reduction on Copper

**DOI:** 10.1002/anie.202104114

**Published:** 2021-06-15

**Authors:** Hongyu An, Longfei Wu, Laurens D. B. Mandemaker, Shuang Yang, Jim de Ruiter, Jochem H. J. Wijten, Joris C. L. Janssens, Thomas Hartman, Ward van der Stam, Bert M. Weckhuysen

**Affiliations:** ^1^ Inorganic Chemistry and Catalysis Institute for Sustainable and Circular Chemistry Utrecht University Universiteitsweg 99 3584 CG Utrecht The Netherlands

**Keywords:** copper, electrocatalysis, in situ, Raman spectroscopy

## Abstract

The electrocatalytic carbon dioxide (CO_2_) reduction reaction (CO_2_RR) into hydrocarbons is a promising approach for greenhouse gas mitigation, but many details of this dynamic reaction remain elusive. Here, time‐resolved surface‐enhanced Raman spectroscopy (TR‐SERS) is employed to successfully monitor the dynamics of CO_2_RR intermediates and Cu surfaces with sub‐second time resolution. Anodic treatment at 1.55 V vs. RHE and subsequent surface oxide reduction (below −0.4 V vs. RHE) induced roughening of the Cu electrode surface, which resulted in hotspots for TR‐SERS, enhanced time resolution (down to ≈0.7 s) and fourfold improved CO_2_RR efficiency toward ethylene. With TR‐SERS, the initial restructuring of the Cu surface was followed (<7 s), after which a stable surface surrounded by increased local alkalinity was formed. Our measurements revealed that a highly dynamic CO intermediate, with a characteristic vibration below 2060 cm^−1^, is related to C−C coupling and ethylene production (−0.9 V vs. RHE), whereas lower cathodic bias (−0.7 V vs. RHE) resulted in gaseous CO production from isolated and static CO surface species with a distinct vibration at 2092 cm^−1^.

## Graphical abstract

Time‐resolved surface‐enhanced Raman spectroscopy (TR‐SERS) reveals the dynamics of the CO_2_ reduction reaction over Cu surfaces with a sub‐second time resolution of 717 ms per spectrum. Anodic treatment and subsequent reduction result in a highly active surface for both SERS and ethylene production within 7 s. A dynamic CO intermediate below 2060 cm^−1^ correlates with CO‐CO coupling and ethylene formation, and a static CO intermediate with a vibration at 2092 cm^−1^ relates to CO production.

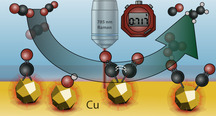

## Introduction

Copper (Cu) is a unique metal due to its outstanding ability to produce ethylene and other C_2+_ products in the electrocatalytic CO_2_ reduction reaction (CO_2_RR).^[1]^ However, the exact reaction mechanism for C_2+_ products is complex and still debated in literature, since multiple electron‐ and proton‐transfer steps are involved that need to occur in a concerted manner.^[2]^ Both the structure of the electrode surface and the chemistry of surface intermediates are considered to be important performance‐deciding factors.^[3]^ Recent research on oxide‐derived Cu electrocatalysts and pulsed‐electrolysis discovered that oxide species covering Cu surfaces are reduced under cathodic bias, which led to improved CO_2_RR activity ascribed to the formation of an activated electrode through surface reconstructions.^[4]^ Adsorbed carbon monoxide (CO) at the catalyst surface has distinct optical signatures, and is usually considered to play a crucial role towards hydrocarbon (e.g., ethylene and methane) formation during CO_2_RR on Cu.[^3d^, ^4b^, ^5^] Through steady‐state vibrational spectroscopy studies, different adsorption modes of CO at the electrode surface have been elucidated, such as CO adsorbed on terrace and defect sites, and bridged CO.[^5d^, ^6^] However, many details about the dynamic surface chemistry of CO intermediates and C−C coupling, which are considered crucial for achieving C_2+_ products,^[7]^ remain unclear. Reconstruction of the catalyst surface, as well as formation and coupling of CO intermediates, may happen on a timescale near or below one second depending on reaction conditions. Therefore, in situ time‐resolved spectroscopic techniques are necessary to follow the evolution of surface reconstruction and adsorbed species in real‐time, in order to gain more insight into the reaction mechanisms.[^6a^, ^8^]

In situ vibrational spectroscopy is a paramount technique for studying the steady‐state chemistry of CO surface species and intermediates on catalyst surfaces.[^5d^, ^5g^, ^9^] Due to the low scattering and absorbing cross sections of water, in situ Raman spectroscopy has seen many successful applications in aqueous environments.[^4c^, ^5f^, ^5g^, ^10^] Compared to infrared spectroscopy (IR), Raman spectroscopy can also easily collect vibrational signal in the low‐wavenumber region, where valuable information about catalyst structure is usually observed (e.g., surface oxide species).^[11]^ However, extended acquisition times are typically required to obtain good signal/noise ratios due to the low Raman scattering probability (in the order of several minutes), which results in a poor time resolution. Surface‐enhanced Raman spectroscopy (SERS) can be exploited to boost the sensitivity towards adsorbed species through surface modifications at the nanoscale, enabling shorter acquisition times.^[12]^ In recent years, SERS has already seen many applications in catalysis research.[^11c^, ^13^] Interestingly, Cu is known to exhibit strong SERS activity in the near infrared excitation wavelength,[^13c^, ^13f^] next to its unique electrocatalytic abilities for C−C coupling and C_2+_ product formation.

In this work, we take advantage of these two characteristics of Cu, which acts both as an active CO_2_RR electrocatalyst and SERS‐active substrate in our experiments, to achieve sub‐second in situ time‐resolved SERS (TR‐SERS) under CO_2_RR conditions. This unique combination enabled us to investigate the dynamic surface reconstruction of Cu, as well as chemical processes of adsorbed CO species on polycrystalline Cu electrodes during CO_2_RR. Our experiments reveal that anodic treatment (1.55 V, all biases in this manuscript are vs. the reversible hydrogen electrode, RHE) and subsequent reduction (<−0.4 V vs. RHE) roughens the electrode surface, resulting in hotspots for enhanced SERS activity, as well as a fourfold increase in CO‐coupling efficiency towards C_2+_ products on Cu‐based electrodes (at −1.0 V vs. RHE). The time‐ and potential‐dependent behavior in our TR‐SERS experiments reveals a dynamic CO surface intermediate with a distinct vibration below 2060 cm^−1^, which only appears for cathodic biases below −0.8 V vs. RHE. Selectivity measurements suggest that this dynamic CO intermediate is correlated to the production of ethylene at a cathodic bias of −0.9 V vs. RHE. At a cathodic bias of −0.7 V vs. RHE, the Raman spectra are dominated by a static vibrational signature at 2092 cm^−1^, which is ascribed to CO adsorbed on undercoordinated sites, resulting in gaseous CO production. Our results display that CO_2_RR and their intermediates are dynamic, and showcase the need for improved time‐resolved in situ spectroscopic investigations down to milliseconds in order to investigate the reaction kinetics in great detail.

## Results and Discussion

**Figure 1:**
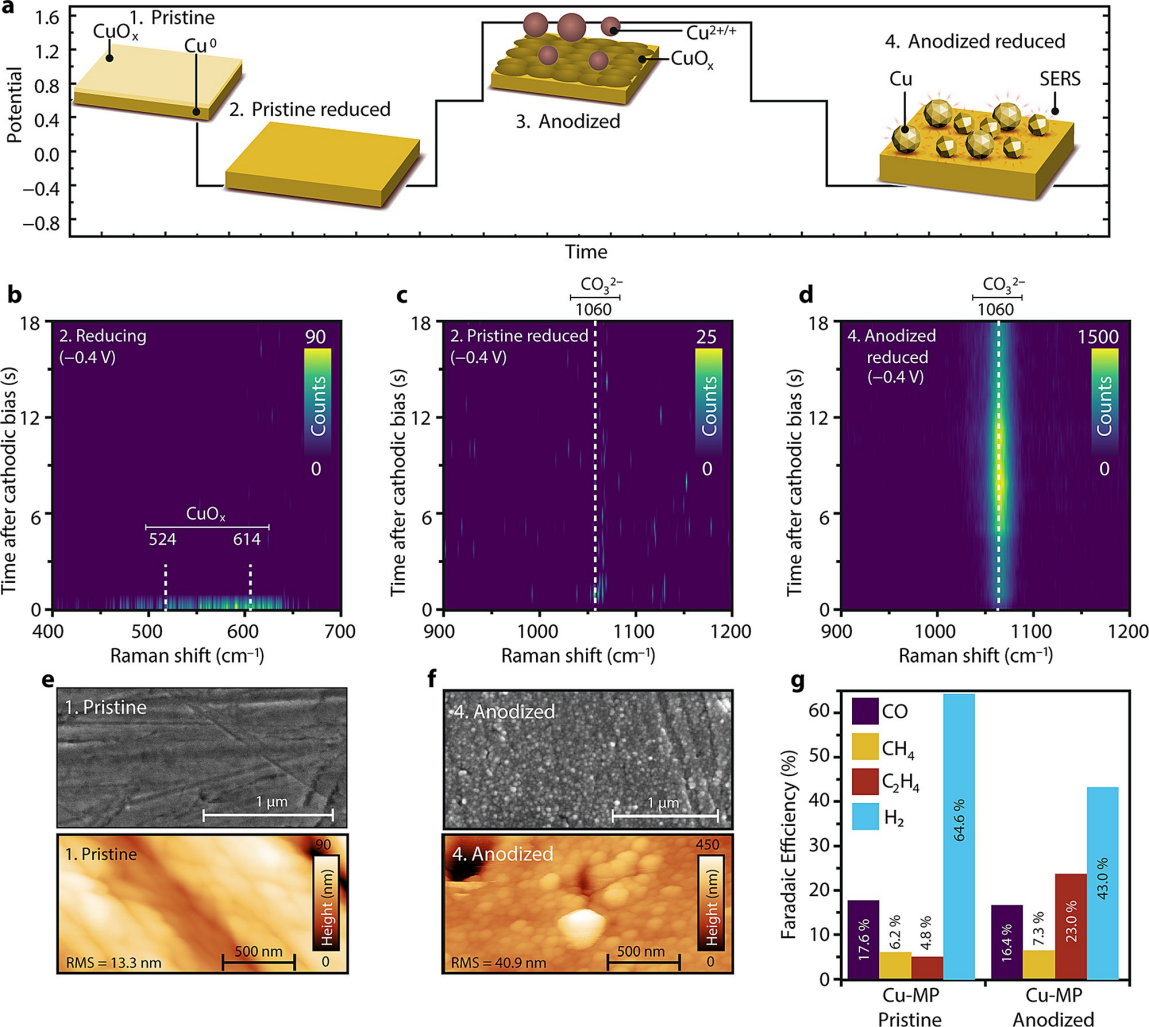
a) Potential pulsing procedure and related states of the Cu‐MP electrode used in this Figure. b) Spectral heatmap from TR‐SERS measurements of pristine Cu‐MP during the first reduction step in the copper oxide region (Raman shift between 400–700 cm^−1^), indicating rapid removal of surface oxide species, and c) TR‐SERS heatmap of pristine reduced Cu‐MP in the carbonate region (Raman shift between 900–1200 cm^−1^), where no obvious bands are observed, indicating a poor SERS effect. d) TR‐SERS heatmap of anodized Cu‐MP in the carbonate region (between 900–1200 cm^−1^) during reduction, displaying a strong carbonate vibration at 1060 cm^−1^, highlighting the strongly enhanced Raman signal after anodic treatment and subsequent reduction. e) SEM (top) and AFM (bottom) images of pristine Cu‐MP and f) anodized Cu‐MP, showing the surface roughening and nanoparticle formation after anodic treatment. g) FE of pristine and anodized Cu‐MP during CO_2_RR at −1.0 V. The color bars of the heatmaps are based on photon counts of Raman spectra. Electrolyte: flowing CO_2_‐saturated 0.1 M KHCO_3_, pH 6.8. Raman spectra interval: 717 ms.

**In situ TR‐SERS at −0.4 V: anodization leads to formation of “hotspots”**. Mechanically polished polycrystalline Cu (referred to as Cu‐MP hereafter)^[14]^ is used as working electrode in our in situ electrochemical Raman cell (three electrode cell, glassy carbon counter electrode, Ag/AgCl reference electrode, CO_2_‐saturated 0.1 M KHCO_3_ aqueous electrolyte, pH 6.8; Supporting Information, Figure S1). We have used both glassy carbon and Pt as counter electrode, in order to exclude possible Pt contamination on the working electrode.^[15]^ No apparent differences were observed (for a comparison see the Supporting Discussion), and the data with glassy carbon as counter electrode is used in the main text of this manuscript. The X‐ray Diffraction (XRD) pattern (Figure S2) shows that the Cu lattice is mainly (100) oriented without indication of surface oxide species.^[1c]^ We designed a potential pulsing experiment (Figure 1 a) to study the time‐dependent SERS intensity from Cu‐MP and the effect of anodic treatment on the surface structure. By continuously collecting spectra at a rate of 717 ms per in situ Raman spectrum we obtained spectral heatmaps, which allowed us to dynamically follow the time‐resolved behavior of the Raman signal. A weak Raman signal of surface oxide can be observed with steady‐state Raman spectroscopy of pristine Cu‐MP before reduction, evidenced by two broad bands at 524 cm^−1^ and 614 cm^−1^ (Figure 1 b).[^6^, ^13c^] These bands, assigned to Cu oxide (CuO_*x*_) surface species, disappear within one second after the onset of −0.4 V reducing potential, in accordance with the cyclic voltammetry (CV) results (Figure S3), evidencing the stripping of surface oxide species,^[17]^ thereby exposing reduced and activated Cu surface for CO_2_RR. No obvious peaks can be observed in the carbonate region (900–1200 cm^−1^, Figure 1 c) for the pristine electrode, suggesting its poor Raman enhancement after reduction. This also suggests that TR‐SERS may not be suitable for ideal, well‐defined Cu facets, which have poor intrinsic Raman signal enhancement. External signal enhancement through Shell‐Isolated Nanoparticles is necessary to study flat Cu electrodes.^[9b]^ After performing an anodic treatment at 1.55 V for 120 s, the Raman signal associated with surface oxide species increases in signal/noise ratio, but again disappears within a second during subsequent reduction at −0.4 V (Figure S4). In addition to the disappearance of the CuO_*x*_ Raman signal, a clear peak at 1060 cm^−1^ is observed one second after the onset of a sufficient cathodic bias (Figure 1 d). This signal is assigned to adsorbed carbonate species (CO_3_^2−^) based on experiments[^13d^, ^18^] and theoretical calculations on oxide‐ derived Cu electrodes.^[4b]^ The signal collected in the bulk electrolyte solution (Figure S5) is much weaker than on anodized and reduced Cu‐MP surface, and it mainly comes from dissolved bicarbonate (HCO_3_^−^, at 1012 cm^−1^),^[18]^ and no CO_3_^2−^ peak (at 1060 cm^−1^) could be detected in the solution. This shows that the HCO_3_^−^ electrolyte ions are rapidly converted into CO_3_^2−^ during the reduction of surface CuO_*x*_ species. We attribute the formation of CO_3_^2−^ in the initial stages after cathodic bias onset to the deprotonation of HCO_3_^−^ due to the depletion of protons during surface oxide/hydroxide reduction and hydrogen evolution reaction (HER). The rapid appearance of the carbonate band after the oxide stripping suggests that the reduced surface of anodized Cu‐MP is highly SERS‐active.

The crucial role of anodization and subsequent reduction on the strong Raman enhancement effect is further evidenced by the scanning electron microscopy (SEM) and atomic force microscopy (AFM) images of pristine (Figure 1 e) and anodized Cu‐MP (Figure 1 f). The pristine Cu‐MP electrode is rather smooth, but also displays grooves as a result of mechanical polishing (Figure 1 e). After anodic treatment and subsequent reduction, nanoparticles can be observed on the electrode surface (Figure 1 f). AFM results show that most of the nanoparticles formed after anodic treatment are between 50 to 150 nm in size (Figure S6), comparable with previous reports about SERS‐active nanoparticles.[^13c^, ^13e^, ^19^] Electrochemical capacitance tests (Figure S7) reveal that the double layer electrochemical capacitance of Cu‐MP increases by a factor of eight after anodization and subsequent reduction, indicating an increase in surface roughness. An increase in surface roughness is also clearly observed in the AFM measurements, in which the root mean square roughness (RMS) of the electrodes increases from 13 nm to 41 nm after anodic treatment (Figure 1 e,f; Figure S6). We also recorded a live video of a Cu‐MP electrode during the entire potential cycling experiment with an optical microscope, which also indicates surface roughening due to the anodic treatment (video link and selected frames shown in Figure S8).

Besides the increased SERS effect that we have observed, anodic treatment has also been applied to increase the faradaic efficiency (FE) and product selectivity toward C_2+_ products during CO_2_RR over Cu electrodes.^[20]^ Product analysis of pristine and anodized Cu‐MP using an H‐Cell (Figure S9) is displayed in Figure 1 g. The reaction potential is set at −1.0 V, which is a standard in literature for measuring ethylene formation on Cu electrodes.[^3f^, ^16^] For both samples, the major products reduced from CO_2_ are methane, CO and ethylene. The FE of Cu‐MP towards ethylene improved fourfold after anodization (23.0 % compared to 4.8 %). This reveals that C−C coupling of CO intermediates are facilitated on the roughened anodized Cu‐MP surface.[^3a^, ^3b^, ^16^] This is in line with the improved SERS enhancement after anodization, suggesting that surface “hotspots” for SERS enhancement and active sites for CO coupling are simultaneously created during nanoparticle formation.

**Figure 2:**
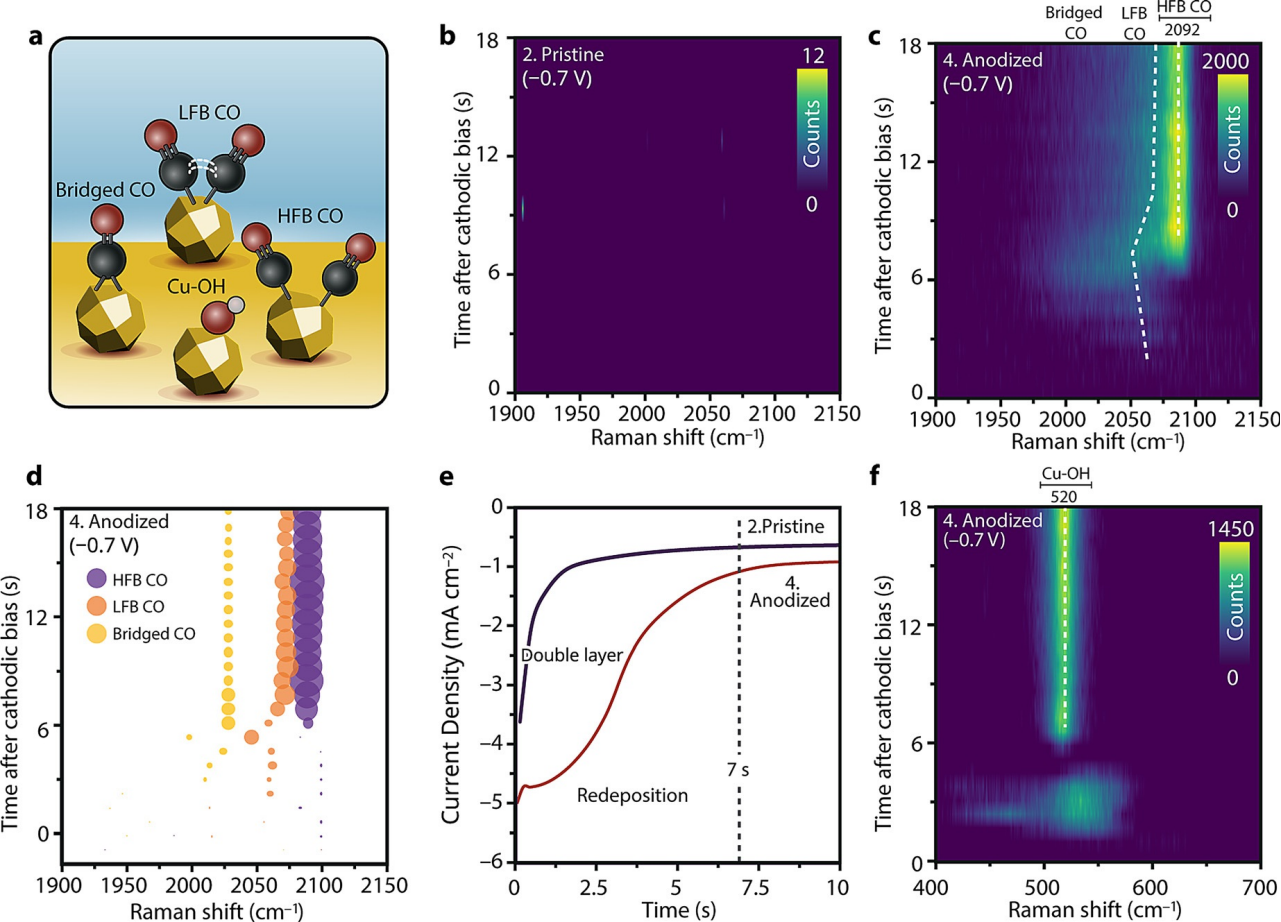
a) Surface species corresponding to the Raman signals observed in this Figure. b) TR‐SERS heatmap of pristine Cu‐MP during reduction at −0.7 V in the CO region (Raman shift between 1900–2150 cm^−1^). c) TR‐SERS heatmap of anodized Cu‐MP during reduction at −0.7 V in the CO region (Raman shift between 1900–2150 cm^−1^), showing the dynamic behavior of adsorbed CO. d) Fitted result of (c), allowing for deconvolution of the Raman spectra into three bands: high‐frequency band CO (HFB, blue bubbles), low‐frequency band CO (LFB, orange bubbles) and bridged CO (yellow bubbles). Bubble positions show time and Raman shift, and bubble sizes are proportional to peak intensities. The color bars of the heatmaps are based on the photon counts of the Raman spectra. e) Chronoamperometry (CA) curve of pristine and anodized Cu‐MP during reduction at −0.7 V in the first 10 s (full data is shown in Figure S11), showing the current associated with redeposition of leached Cu after anodic treatment. f) TR‐SERS heatmap of anodized Cu‐MP during reduction at −0.7 V in the copper oxide and Cu‐OH region (Raman shift between 400–700 cm^−1^), showing the formation of Cu‐OH (peak at 520 cm^−1^) after 7 s of cathodic bias. Electrolyte: flowing CO_2_‐saturated 0.1 M KHCO_3_ aqueous solution, pH 6.8. Raman spectra interval: 717 ms.

**In situ TR‐SERS at −0.7 V: CO intermediates and local alkalinity**. It is generally accepted that adsorbed CO is a key intermediate in CO_2_RR towards hydrocarbon products on Cu electrocatalyst surfaces.[^3d^, ^5^] To investigate the reaction mechanism, we performed in situ TR‐SERS measurements to investigate the dynamics of CO intermediates. The potential pulsing procedure for the surface treatment and subsequent reduction is the same as in Figure 1 a, except for the reduction potentials (set at −0.7 V). The assignment of observed species is depicted in Figure 2 a.

TR‐SERS heatmaps of the stretching vibration of adsorbed CO (in the 1900–2100 cm^−1^ region) on pristine and anodized Cu‐MP at −0.7 V are shown in Figure 2 b,c, respectively. On pristine Cu‐MP, no SERS signal can be detected in the CO region (Figure 2 b). For anodized Cu‐MP, the CO Raman signal intensity increases dramatically, which allows us to follow the dynamics of the CO intermediates with sub‐second time resolution (Figure 2 c). By fitting the Raman spectra, we deconvolute the spectra of anodized Cu‐MP in the C=O stretching range into three peaks, based on the previous work by Gunathunge et al.[^6a^, ^6b^, ^8a^, ^20^] (Figure 2 d): (1) bridged CO at ≈2030 cm^−1^, (2) low‐frequency band linear CO (LFB‐CO) at ≈2060 cm^−1^, and (3) high‐frequency band linear CO (HFB‐CO) at ≈2095 cm^−1^. The first species to be observed in this region are two wide peaks centered at 2058 cm^−1^ (LFB‐CO), which appear within 2 s after the onset of cathodic bias. The LFB‐CO is typically associated with adsorbed CO on top of terrace‐like sites according to previous reports on well‐defined facets.[^6a^, ^20^] This LFB‐CO peak shifts towards lower Raman shifts over time during the first ≈7 s of cathodic bias onset. After this initial shift of LFB‐CO, a sharper peak at 2092 cm^−1^ becomes visible after 7 s, which becomes the dominating species. The HFB‐CO peak at 2092 cm^−1^ is ascribed to adsorbed CO on isolated defect‐like sites, based on observations from previous in situ measurements on well‐defined systems.^[21]^ The LFB‐CO peak also shifts to 2072 cm^−1^ and remains as a weak shoulder next to the HFB‐CO peak after ca. 7 s. The peak positions and intensities of both HFB‐CO and LFB‐CO remain stable after ≈9 s of cathodic bias, up to 20 min of cathodic bias (Figure S10).

The chronoamperometry (CA) data of pristine and anodized Cu‐MP samples is shown in Figure 2 e (complete 120 s CA data in Figure S11). In addition to the charging behavior of the electrochemical double layer in both samples, an additional peak is observed for anodized Cu‐MP (Figure 2 e), related to the formation of nanostructures on the surface.^[22]^ This additional peak is ascribed to redeposition of dissolved Cu^2+^/CuO_*x*_(OH)_*y*_ species from solution, which were created during the anodic treatment.[^22^, ^23^] Most of the redeposition process is complete after ca. 7 s of reduction, which matches with the time scale of the observed transition of the adsorbed CO species from LFB‐dominating to HFB‐dominating (Figure 2 b,c). TR‐SERS results of anodized Cu‐MP in the oxide region (400–700 cm^−1^, Figure 2 f) show that after rapid oxide stripping within the first second, a new peak at 520 cm^−1^ emerges after ca. 7 s of cathodic bias, which is assigned to adsorbed Cu‐OH at the electrode surface.[^11c^, ^13c^, ^16^, ^24^] This peak originates from the increase of local alkalinity (and increase in the local hydroxide concentration), which stems from the depletion of protons near the electrode surface during HER.[^16^, ^18^, ^25^] These results suggest that during the first 7 seconds after the onset of −0.7 V bias, the main reduction process is the redeposition of Cu species leached into the electrolyte, which results in the formation of nanostructures. After 7 s, the HER and CO_2_RR processes dominate, and the increase of local alkalinity near the electrode surface results in the accumulation of Cu‐OH as major species. Furthermore, surface hydroxide has also been reported to be a promoter for CO_2_RR.[^8b^, ^26^] These restructuring processes at the electrode surface in turn changed the chemical state of the adsorbed CO species, resulting in the observed dynamic nature of the bands associated with adsorbed CO in the initial phase (first 7 s) of the reaction (Figure 2 c). Compared to IR, the ability of (TR‐)SERS to collect vibrational features at low wavenumbers (<800 cm^−1^) allows us to correlate the redeposition, local alkalinity and CO intermediates in a time‐resolved manner.

**Figure 3:**
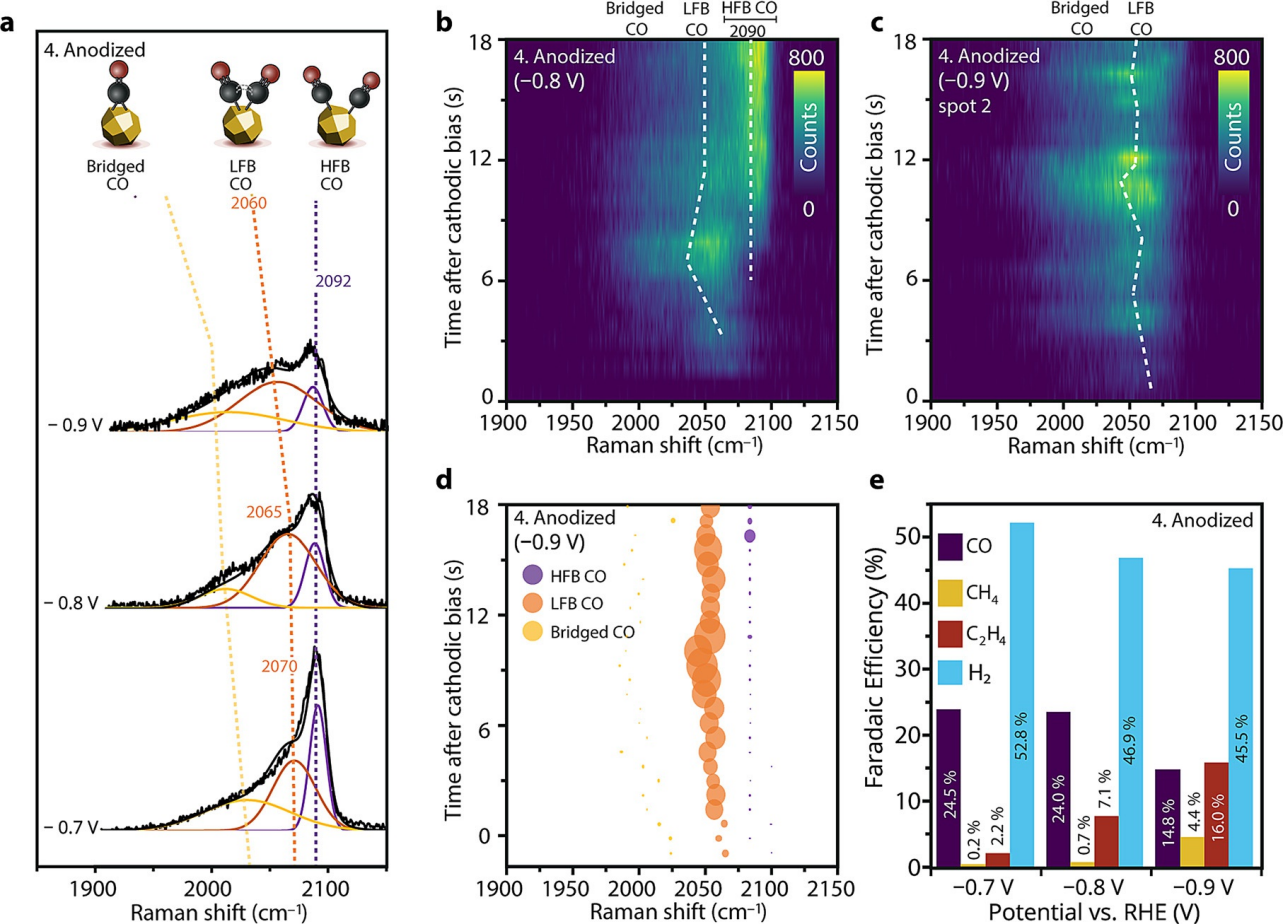
a) Comparison of steady‐state Raman spectra (15 min after reduction) of anodized Cu‐MP during reduction at −0.7 V, −0.8 V and −0.9 V. Collection time is 5 s. b) TR‐SERS heatmap of anodized Cu‐MP during reduction at −0.8 V in the CO region (Raman shift between 1900–2150 cm^−1^). After 7 s, an intense high‐frequency band CO (HFB‐CO) is observed. c) TR‐SERS heatmaps of anodized Cu‐MP during reduction at −0.9 V in the CO region (Raman shift between 1900–2150 cm^−1^), showing that the low‐frequency band CO (LFB‐CO) is highly dynamic. d) Fitted result of (c), showing the increased contribution of LFB‐CO to the spectrum at −0.9 V. Bubble position shows time and Raman shift, and bubble size is proportional to peak intensity. e) FE of anodized Cu‐MP during CO_2_RR at −0.7 V, −0.8 V and −0.9 V. The color bars of the heatmaps are based on photon counts of Raman spectra. Electrolyte: CO_2_‐saturated 0.1 M KHCO_3_ with CO_2_ bubbling, pH 6.8. Raman spectra interval for TR‐SERS: 717 ms.

**In situ TR‐SERS beyond −0.8 V: potential‐dependent dynamic CO and product selectivity**. The product distribution of CO_2_RR on Cu is known to strongly depend on cathodic potential.[^1a^, ^1b^, ^1c^, ^1d^, ^3g^] To elucidate the relationship between adsorbed CO species and CO_2_RR selectivity, we performed TR‐SERS on anodized Cu‐MP at −0.8 V and −0.9 V (Figure 3). The potential pulsing protocols are similar to Figure 1 a, except for the more cathodic reduction biases of −0.8 and −0.9 V. Steady‐state in situ Raman spectra (collected ca. 15 min after cathodic bias onset) at all the three potentials (Figure 3 a) show that the HFB‐CO peak positions are at slightly different positions for varying potentials (2093, 2090, and 2086 cm^−1^ at, respectively −0.7, −0.8, and −0.9 V). The relative intensity of HFB‐CO compared to LFB‐CO decreases at more cathodic potential, while an increase in relative peak intensity of LFB‐CO (compared to HFB‐CO) is observed at more cathodic potentials (Figure 3 a). Meanwhile, the LFB‐CO peak also displays more drastic potential dependent shift to lower Raman wavenumbers, from 2070 cm^−1^ at −0.7 V to ≈2050 cm^−1^ at −0.9 V. This is caused by the electrochemical Stark effect,^[5e]^ which results from the interaction between the applied electric field and top‐oriented adsorbed CO molecules, resulting in a potential dependent shift.

During TR‐SERS measurements at different applied potentials, the formation and shift towards lower Raman shift of initial LFB‐CO at −0.8 V (Figure 3 b; Figure S12) is similar to the scenario at −0.7 V (Figure 2 c). Furthermore, the spectrum changes from LFB‐CO dominant to HFB‐CO dominant after ca. 7 s, similar to the behavior at −0.7 V. This further supports the notion that the surface adsorbed CO is affected by local alkalinity and the redeposition of Cu nanoparticles in the first 7 s, also at −0.8 V. At −0.9 V, LFB‐CO exhibits a very dynamic peak shifting behavior, as shown in Figure 3 c, and the initially formed LFB‐CO does not transform into HFB‐CO after ≈7 s. Instead, it exhibits rapid peak position shifting between 2040 and 2060 cm^−1^ (deconvoluted peak positions shown in Figure 3 d) up to 20 min after cathodic bias onset (Figure S13). Small variations in total signal intensity are mostly caused by the rapid formation of bubbles due to gaseous products at these overpotentials. However, the shift of the LFB‐CO peak position as a function of time suggests that it is highly dynamic in nature and actively involved in surface reactions, which is expected for CO intermediates that are involved in complex reactions such as ethylene formation. The HFB‐CO can still be spotted in some observations at −0.9 V, but with weaker intensity compared to the experiment at less cathodic biases, with dynamic LFB‐CO at ≈2050 cm^−1^ being dominant (Figure S14). The potential‐dependent FE of anodized Cu‐MP during CO_2_RR is shown in Figure 3 e. The clear trend that CO FE decreases (from 24.5 % to 14.8 %), while that of ethylene increases (from 2.2 to 16.0 %) when the applied potential is varied from −0.7 V to −0.9 V suggests that the dynamic LFB‐CO species (at 2050 cm^−1^ Raman shift at applied potential of −0.9 V) observed with our in situ TR‐SERS experiments are related to CO‐CO coupling and ethylene production, whereas the presence of the HFB‐CO peak at 2092 cm^−1^ is more related to gaseous CO production.[^3d^, ^27^] Since the GC product analysis (injection every 4 min) is a lot slower than our TR‐SERS (sub‐second collection of Raman spectra), we also extended the TR‐SERS time regime to 20 min (with a Raman spectrum every 10 s) in order to cover the GC detection window, and to analyze the stability or dynamics of the observed features on those extended timescales (Figures S10 and S13). As discussed above, we observe dynamic behavior of LFB‐CO at ethylene‐dominating potentials of −0.9 V on the timescale of 20 min, whereas the HFB‐CO SERS signal remains stable at −0.7 V and −0.8 V over the course of the experiments. Furthermore, the Cu‐OH signal is also constant during 20 min of cathodic bias (Figure S15), indicating that the surface of the electrode is stable during our TR‐SERS experiments. This further corroborates the notion that the dynamic LFB‐CO is related to electrocatalytic reactions at the electrode surface at more cathodic bias, and not to dynamics in the surface structure as a function of time.

The apparent differences in the time‐dependent behavior of LFB‐CO at applied potentials between −0.7 V and −0.9 V imply that the LFB‐CO intermediates play a different, potential‐dependent role in CO_2_RR. LFB‐CO under ethylene‐producing potentials of −0.9 V has a lower Raman shift than under CO‐dominating potentials of −0.7 V (2060 vs. 2070 cm^−1^, respectively). This lower peak position suggests weakened C=O bonds, which would facilitate subsequent CO‐CO coupling steps. The significantly higher tendency towards dynamic peak position shifting of the LFB‐CO at −0.9 V suggests more active chemical nature of LFB‐CO at ethylene‐producing bias, revealing its relation to C−C coupling. In contrast, the position of the HFB‐CO Raman band remains stable at all biases and over longer timescales, implying it has a more static behavior. This leads to the conclusion that this dynamic LFB‐CO below 2060 cm^−1^ at −0.9 V is the active CO intermediate for C_2_ product formation, and cannot be assigned to bridged CO, which is typically observed in an even lower Raman shift region (<2020 cm^−1^). The improved FE towards ethylene after the anodization‐reduction cycle suggests that CO‐CO coupling is related to defect formation. We propose that two different types of defect sites can be produced after the anodization‐reduction cycle, namely isolated defect sites and step‐edge sites. CO on isolated sites gives rise to HFB‐CO, and on step‐edge sites to LFB‐CO. Such step‐edge Cu sites share more similarity to terrace sites than isolated Cu sites, which explains the closer Raman peak position of LFB‐CO to terrace‐CO (usually around 2050 cm^−1^).[^6a^, ^20^] Meanwhile, CO on step‐edge Cu sites has a higher chance of coupling with a neighboring adsorbed CO intermediate compared to isolated Cu sites, facilitating CO‐CO coupling and C_2+_ product formation.

**Figure 4:**
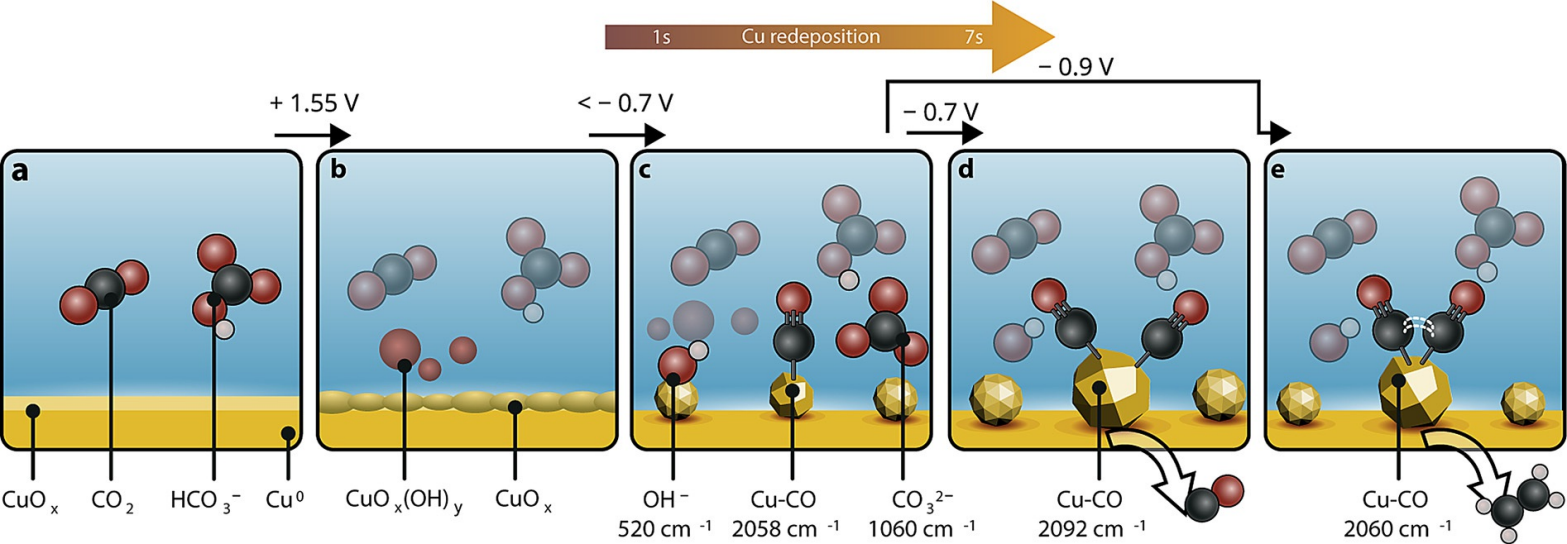
Representation of the observed processes on Cu‐MP during CO_2_RR using in situ TR‐SERS. a) The pristine electrode is rather smooth and contains surface oxide species, but b) anodic treatment (at 1.55 V) roughens the electrode surface and results in leached Cu species in the electrolyte. c) Redeposition of these dissolved Cu species from the electrolyte at potentials <−0.7 V creates nanoparticles at the electrode surface, which results in hotspot formation and an increase in SERS activity. This is followed by the build‐up of local alkalinity. Subsequent CO_2_RR at d) −0.7 V results in CO adsorption on undercoordinated defect‐like sites, and formation of gaseous CO as main product. e) Reduction at a higher cathodic bias (−0.9 V) stabilizes CO at step‐edge sites, thereby inducing C−C coupling and ethylene formation.

We suggest a possible mechanism for improved Raman enhancement and CO_2_RR performance on anodized Cu‐MP based on our in situ TR‐SERS measurements at different applied potentials, as shown in Figure 4. Pristine Cu‐MP is not very SERS active due to the presence of surface oxide and its lack of nanostructures (Figure 4 a), but anodic treatment at 1.55 V vs. RHE (Figure 4 b) and subsequent reduction (Figure 4 c) result in a highly active surface for both SERS and CO_2_RR (fourfold increase in ethylene FE at −1.0 V) due to nanoparticle formation. Furthermore, our results show that the native surface oxide species of anodized Cu‐MP can be stripped within 1 s after cathodic bias onset. In the first 7 s after cathodic bias onset, the main process is the redeposition of leached Cu^2+^/CuO_*x*_(OH)_*y*_ species, creating nanostructures and hotspots for both Raman enhancement and CO_2_RR reaction. After 7 s, the local alkalinity near the electrode surface starts to build up when the cathodic bias is −0.7 V or beyond. At −0.7 V (Figure 4 d), the dimerization of the initial 2058 cm^−1^ terrace LFB‐CO cannot be efficiently triggered, and the LFB‐CO species evolve into stable HFB‐CO on isolated undercoordinated defect‐like sites (Raman peak at ≈2092 cm^−1^). These isolated CO intermediates then desorb as gaseous CO, which is the main CO_2_ reduction product at −0.7 V. This suggests that an isolated defect Cu site stabilizing two adsorbed CO molecules with enough proximity to induce CO‐CO coupling is unlikely. At high cathodic bias (−0.9 V), further reaction of activated LFB‐CO (Raman peak at 2058 cm^−1^) can be triggered, suppressing its conversion toward HFB‐CO and subsequent CO production (Figure 4 e). It has been found that ethylene formation is facilitated on high‐index Cu facets rich in step‐edge sites.[^3b^, ^4g^] Compared to flat pristine Cu‐MP electrode, our anodization‐reduction cycle simultaneously facilitates the formation of defect sites, exhibits increased CO SERS signal intensity and promotes ethylene production. Therefore, we assign this more active and dynamic species at or below 2060 cm^−1^ to CO on step‐edge Cu sites with higher tendency towards CO‐CO coupling, since the coupling between terrace CO molecules would further weaken the CO bond, resulting in the observed lower Raman shift compared to the LFB‐CO at −0.7 V (at 2070 cm^−1^). This assignment is further corroborated by the more dynamic time‐dependent shifting of this CO band (even up to 20 min after cathodic bias onset), which reveals that this intermediate is heavily involved in chemical reactions and facilitates CO‐CO coupling towards ethylene formation (as evidenced by the activity measurements at −0.9 V). This dynamic information of surface reconstruction and surface‐bound intermediates on the sub‐second timescale is often obscured in steady‐state measurements, highlighting the importance of time‐resolved investigations under catalytically relevant conditions.

## Conclusion

We have successfully performed in situ time‐resolved surface‐enhanced Raman spectroscopy (TR‐SERS) during CO_2_ electrochemical reduction on Cu electrodes with sub‐second time resolution. Anodic pretreatment and subsequent reduction of the Cu electrodes create nanostructures via redeposition of dissolved Cu species, which act as “hotspots” for SERS as well as active sites for CO coupling at high cathodic overpotential (−0.9 V vs. RHE). These nanostructures allow for TR‐SERS measurements with sub‐second time resolution and a fourfold improvement in ethylene production (up to 23 % FE at −1.0 V vs. RHE). Our TR‐SERS measurements reveal that surface reconstruction and nanostructure formation happen within the first 7 s after cathodic bias onset, after which an increase in local alkalinity creates a stable Cu‐OH electrode surface. CO adsorbed on isolated undercoordinated defect sites (such as nanoparticle corners and edges) dominates at −0.7 V vs. RHE, characterized by a static Raman band at ≈2092 cm^−1^, and we correlate this more static CO with gaseous CO product desorption. An activated CO intermediate, characterized by a dynamic Raman band at <2060 cm^−1^, dominates at −0.9 V vs. RHE, whose dynamic potential‐ and time‐dependent behavior suggests its tendency towards dimerization and the formation of ethylene at more cathodic bias. Our results demonstrate that in situ TR‐SERS with sub‐second time resolution is a vital technique for achieving dynamic information of surface reactions during CO_2_ electrolysis.

## Conflict of interest

The authors declare no conflict of interest.

## Supporting information

As a service to our authors and readers, this journal provides supporting information supplied by the authors. Such materials are peer reviewed and may be re‐organized for online delivery, but are not copy‐edited or typeset. Technical support issues arising from supporting information (other than missing files) should be addressed to the authors.

Supplementary
