# Supplementary material for: Sub‐Second Time‐Resolved Surface‐Enhanced Raman Spectroscopy Reveals Dynamic CO Intermediates during Electrochemical CO2 Reduction on Copper
Source: Angew Chem Int Ed Engl. 2021 Jun 15;60(30):16576–84. doi: 10.1002/anie.202104114 (PMC8362134; doi:10.1002/anie.202104114)
Supplement: Supplementary file 1 — Supplementary [file ANIE-60-16576-s001.pdf]

Angewandte Chemie
Eine Zeitschrift der Gesellschaft Deutscher Chemiker

Supporting Information

Sub-Second Time-Resolved Surface-Enhanced Raman Spectroscopy Reveals Dynamic CO Intermediates during Electrochemical CO_2 Reduction on Copper

Hongyu An, Longfei Wu, Laurens D. B. Mandemaker, Shuang Yang, Jim de Ruiter, Jochem H. J. Wijten, Joris C. L. Janssens, Thomas Hartman, Ward van der Stam, and Bert M. Weckhuysen**

anie_202104114_sm_miscellaneous_information.pdf

Experimental Details

Chemicals: Copper foil (Cu, purity: 99.9999%, puratonic) was purchased from Alfa Aesar. Potassium bicarbonate ($KHCO_3$, purity >99.7%) was purchased from Fluka. Sulfuric acid (H_2SO_4, AnalaR NORMAPUR® analytical reagent, 95~97%) was purchased from VWR Chemicals. Carbon dioxide gas (CO_2, purity: 99.995%) was purchased from Linde Gas. Polishing powders (5 µm CeO_2 and 1 µm Al_2O_3) were purchased from Bodemschat.

Sample preparation: For the preparation of the Cu-MP sample, the Cu foil was cut into strips, then polished with 5 µm CeO_2 and 1 µm Al_2O_3 polishing powders. Then the sample was sonicated in deionized water (Milli-Q) for 2 mins, immersed in 1 M HNO_3 for 1 min, then sonicated in Milli-Q water for 5 min, and rinsed with flowing deionized water for 5 min.

Sample characterization: X-ray diffraction (XRD) measurements were performed on a Bruker D2 PHASER diffractometer using Co Kα (1.789 Å) radiation. Scanning electron microscopy (SEM) images were obtained on FEI Helios Nanolab G3 with accelerating voltage of 5.0 keV and probe current of 25 pA. Atomic Force Microscopy of the Cu-MP samples was measured using an NT-MDT NTEGRA Spectra with NHCV-Al tips (F = 40 N/m). Post data treatment was done using the open-source software Gwyddion, and consisted of a plane background subtraction and line-by-line correction. The grain size distributions were obtained by manually measuring the diagonal of 150 grains using the "Measure distances" function. Root mean square roughness (RMS) was determined by the "Statistical quantities" feature over the full region of interest (ROI).

Electrochemistry measurements: Cyclic voltammetry (CV) and chronoamperometry (CA) measurements of the samples were conducted using an Ivium compactstat.h10800 potentiostat. All electrochemical experiments were performed in aqueous CO_2-saturated 0.1M $KHCO_3$ solution (pH 6.8) using glassy carbon as counter electrode and leakless Ag/AgCl reference electrode (eDAQ) as reference electrode (scan rate 50 mV/s), unless stated otherwise. The CV scans started from open circuit potential (usually -0.08 V vs. Ag/AgCl) to cathodic direction first in order to study the nature of native surface oxide species. The cathodic endpoint was -1.2 V, and anodic endpoint was set at +0.4 V, both against Ag/AgCl. The CA measurements were conducted between open circuit potential and -1.35 V vs. Ag/AgCl. The electrochemical capacitance test was conducted by varying scanning rate in a potential region where no Faradaic processes take place (0 V to -0.2 V vs. RHE). The potential was converted to RHE using the following formula:

$$E(vs.RHE) = E(vs.Ag/AgCl) + 0.197\,V + 0.0591 * pH$$

Raman spectroscopy measurements: Raman spectra acquisition was performed using a Renishaw InVia Raman microscope and 785 nm excitation laser. A Nikon N40X-NIR water-dipping objective was used for signal collection. The laser power needs to be kept below 1.5 mW to protect the sample from laser damage. A custom Raman *in situ* cell (Fig. S1) was used for Raman signal collection. We would like to note that the cell is not a triphasic flow cell (which includes gas-diffusion electrodes) that are used to achieve high current in many publications. The working electrode still contacts only CO_2-saturated electrolyte, and we choose a moderate flow rate (~2 mL/min) to make the mass transfer condition comparable to an H-Cell. The collection time was 0.5 s for Cu-MP sample. The mechanical shutter of the Raman system requires an additional ca. 0.217 s for operation, resulting

in a time resolution of 0.717 s. A grating with 1200 lines/mm was used for maximum efficiency. This limits the wavenumber coverage of each single TR-SERS collection to ca. 250 cm^{-1}, thus spectra in different potential windows were carried out in different experiments. Anodic treatment (+1.55V vs. RHE for 120 s) is needed before subsequent experiments to achieve SERS activity. The potential was applied through an Autolab PGSTAT 101 potentiostat. All other electrochemistry setups are the same as above mentioned.

Product analysis: The activity evaluation of CO_2RR was conducted in a custom quartz H-cell. A piece of glassy carbon (~3 cm^2) served as counter electrode, and a Ag/AgCl reference electrode (eDAQ) was used for controlling the applied potential. The flow rate of CO_2 was kept at 8.7 sccm. CO, CH_4 and C_2H_4 were quantified using an Interscience online gas chromatography equipped with an FID detector. H_2 was quatified using a TCD detector and Kr as internal calibration gas. The Faradaic Efficiency (FE) was calculated using the following formula:

$$FE\% = \frac{c \cdot f \ \cdot \ F \ \cdot n_i}{i \ \cdot \ V_m \ \cdot 60sec/\min \ \cdot \ 1000000ppm} \times 100\%$$

Where c is the concentration obtained from the GC (in ppm), f is the flow rate of CO_2 gas (in mL/min), F is the Faradaic constant (96485 C/Mol), n_i is the number of electrons transferred to a given product (unitless), i is the average current in 1 minute (A), V_m is the volume of 1 mol gas at reaction temperature and pressure (24451 mL/mol).

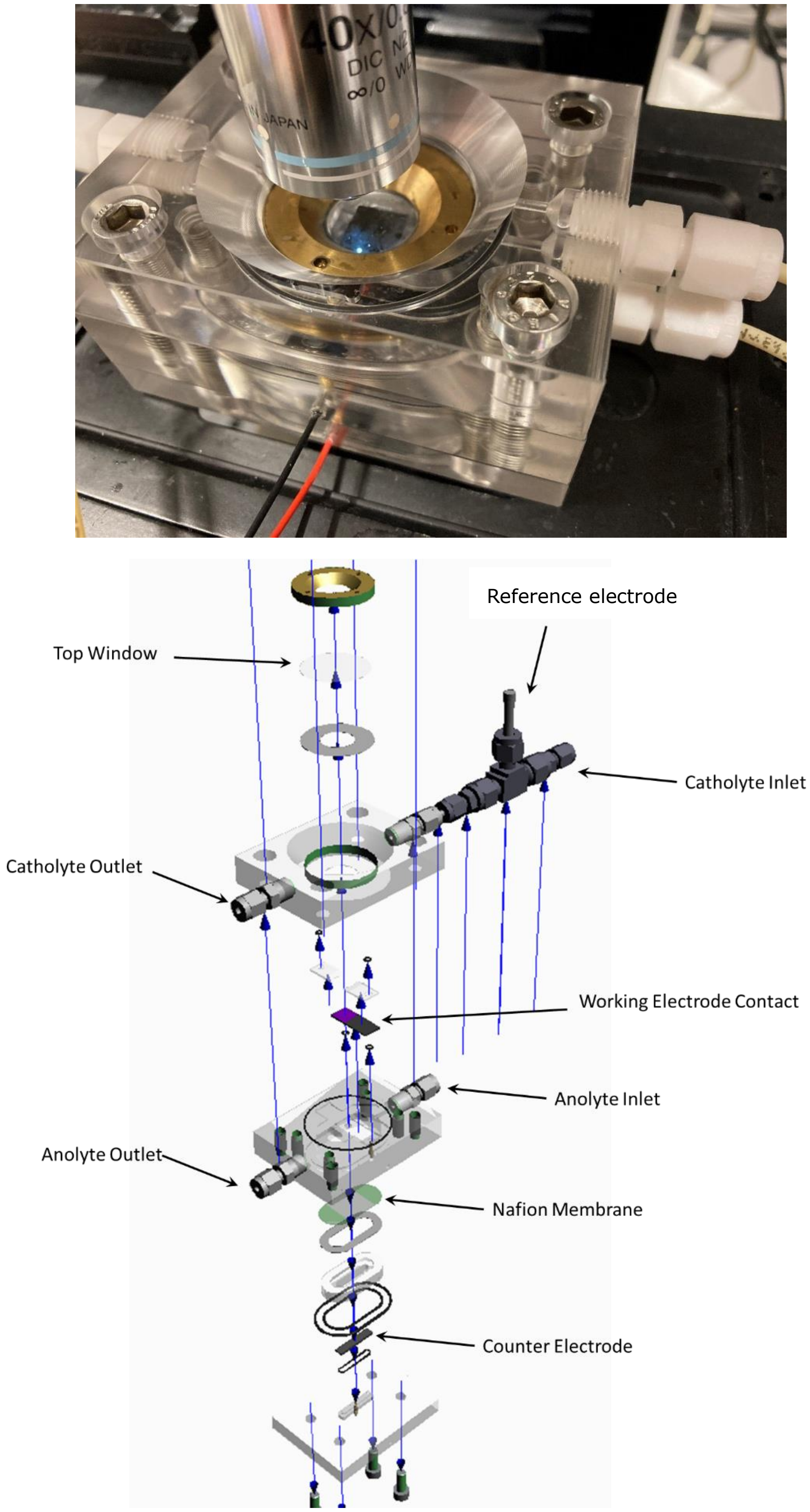

Figure S1: Picture of the customized three electrode *in situ* Raman flow cell.

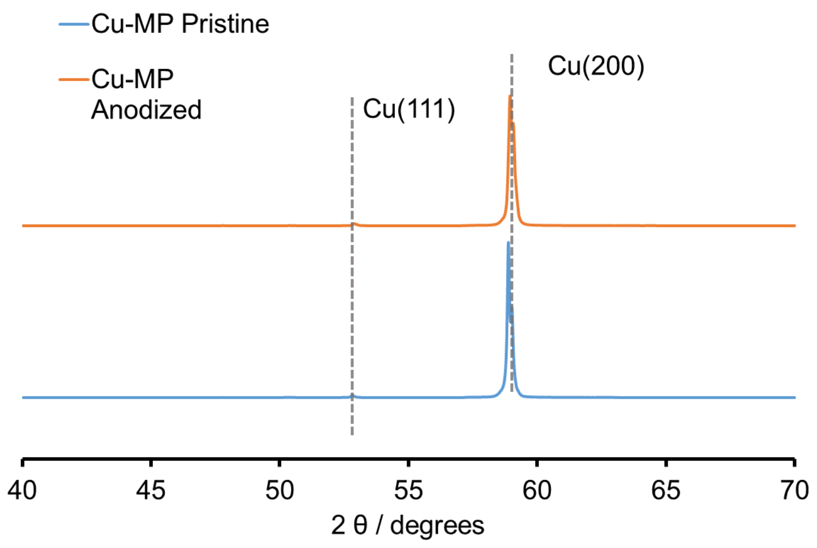

Figure S2: X-ray Diffraction (XRD) pattern of pristine Cu-MP (blue curve) and Cu-MP anodized at +1.55 V for 120 sec and then reduced at -0.4 V for 120 s (Cu-MP anodized).

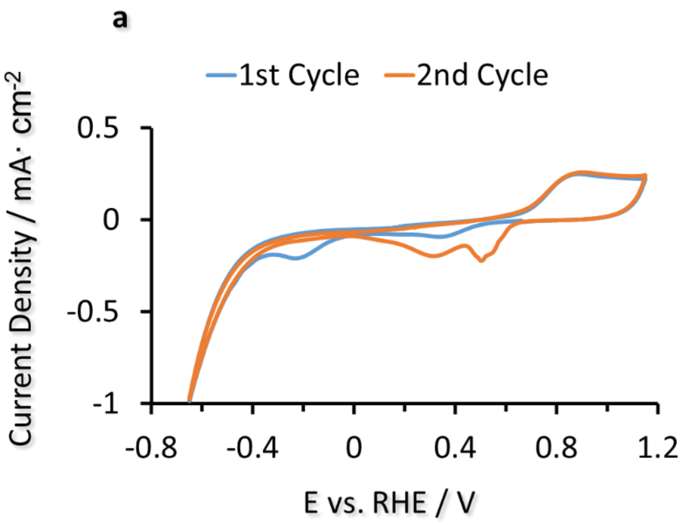

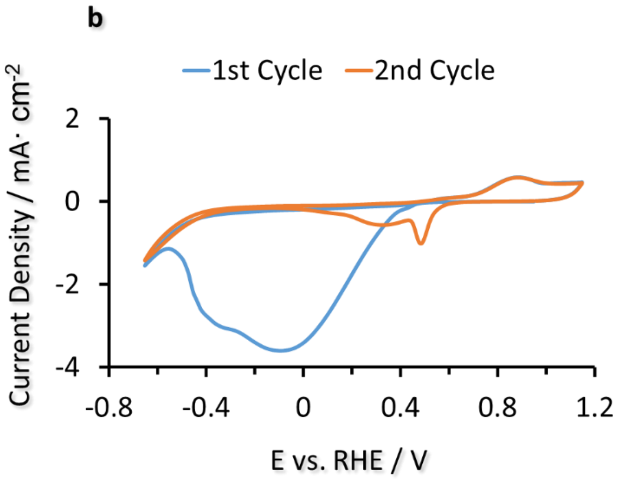

Figure S3: Cyclic voltammetry (CV) curves of (a) pristine Cu-MP and (b) anodized Cu-MP in CO_2-saturated 0.1 M $KHCO_3$, pH = 6.8, scan rate 50 mV/s. Acquired from the same foil.

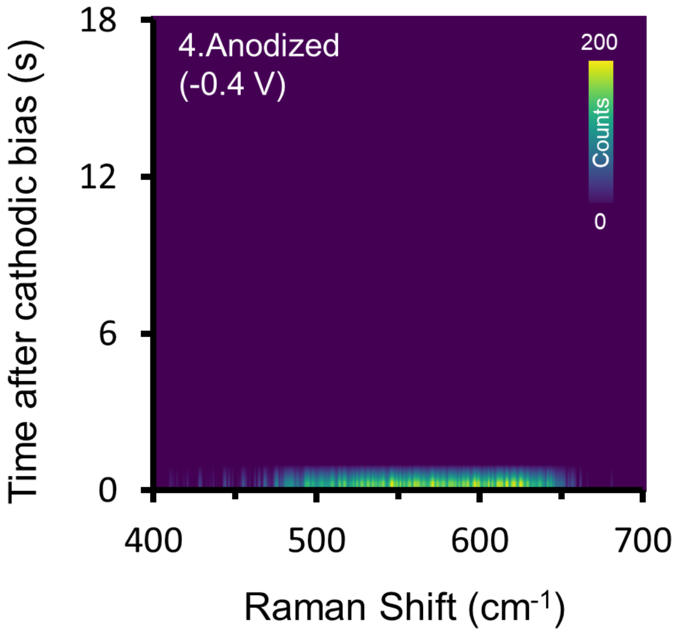

Figure S4: Time-Resolved Surface Enhanced Raman Spectra (TR-SERS) of anodized Cu-MP during reduction at -0.4 V vs. RHE, Raman shift between 400-700 cm^{-1}. Electrolyte: flowing CO_2-saturated 0.1 M $KHCO_3$, pH = 6.8.

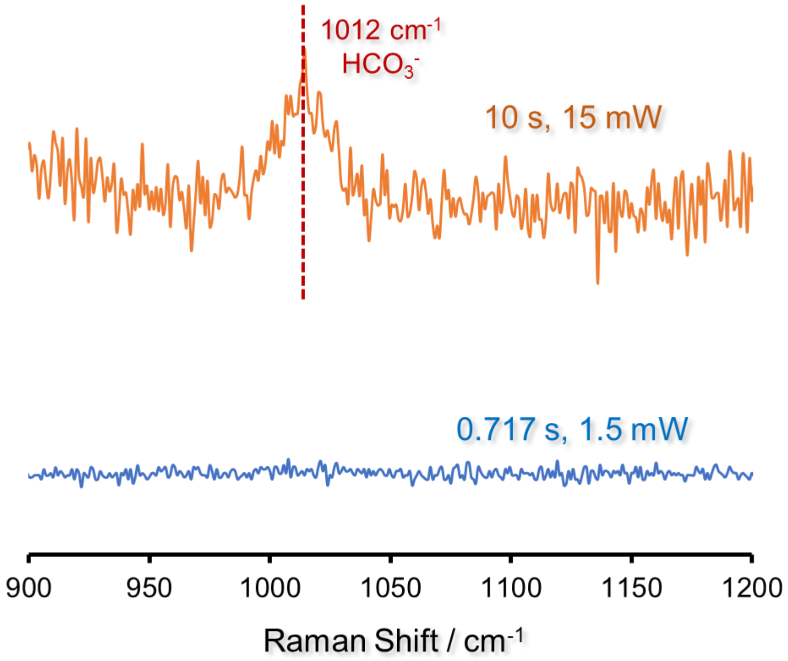

Figure S5: Raman spectra collected when focal point is set in bulk solution, Raman shift between 900-1200 cm^{-1}. Electrolyte: flowing CO_2-saturated 0.1 M $KHCO_3$, pH = 6.8. Laser power and collection time are indicated. The conditions for the blue curve are identical to all TR-SERS measurements.

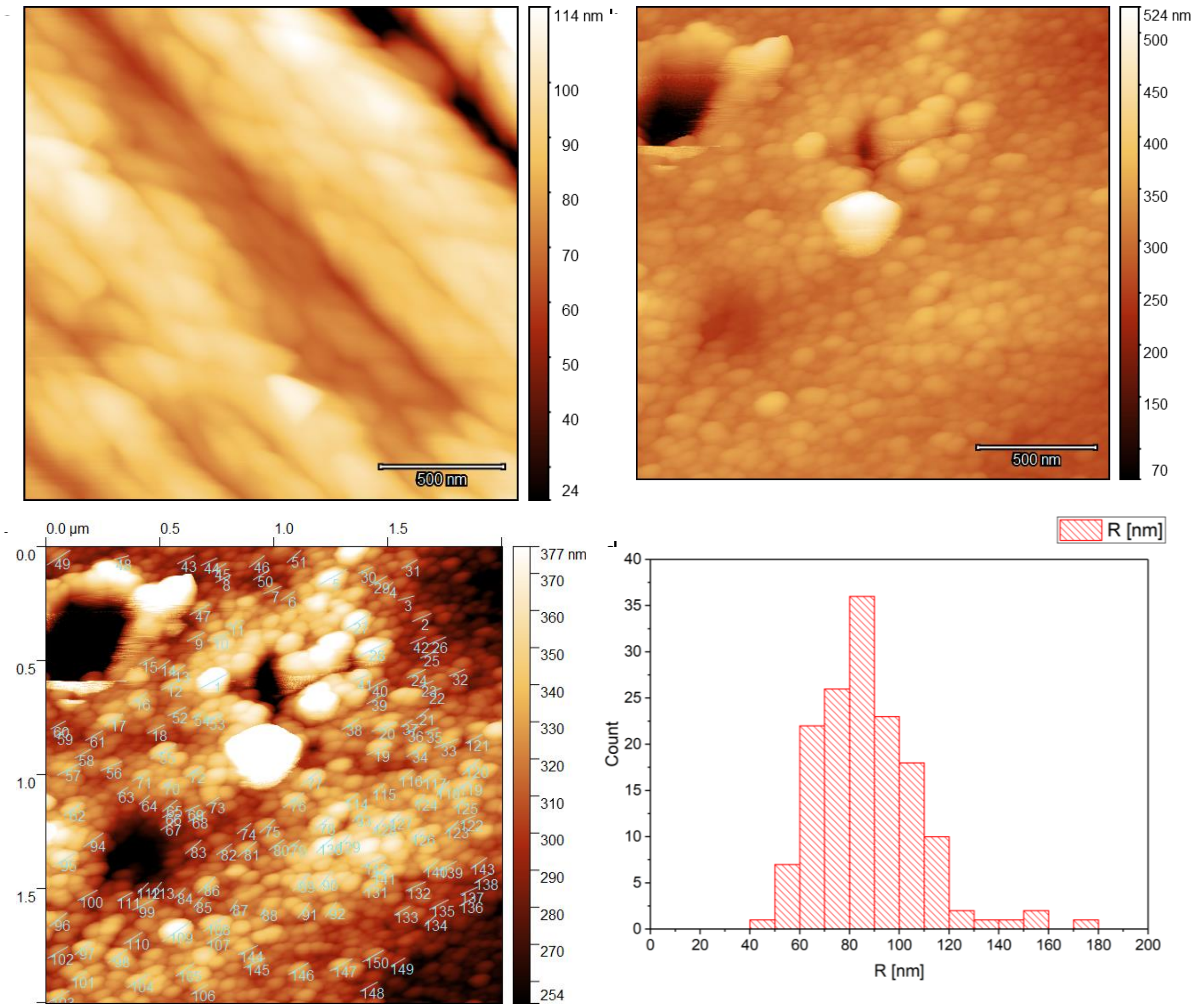

Figure S6: Atomic force microscopy (AFM) images of (a) pristine and (b) anodized Cu-MP electrodes. (c) Nanoparticle size determination and (d) distribution of Cu-MP after anodization at +1.55 V for 120 s and subsequent reduction at -0.4 V for 60 s.

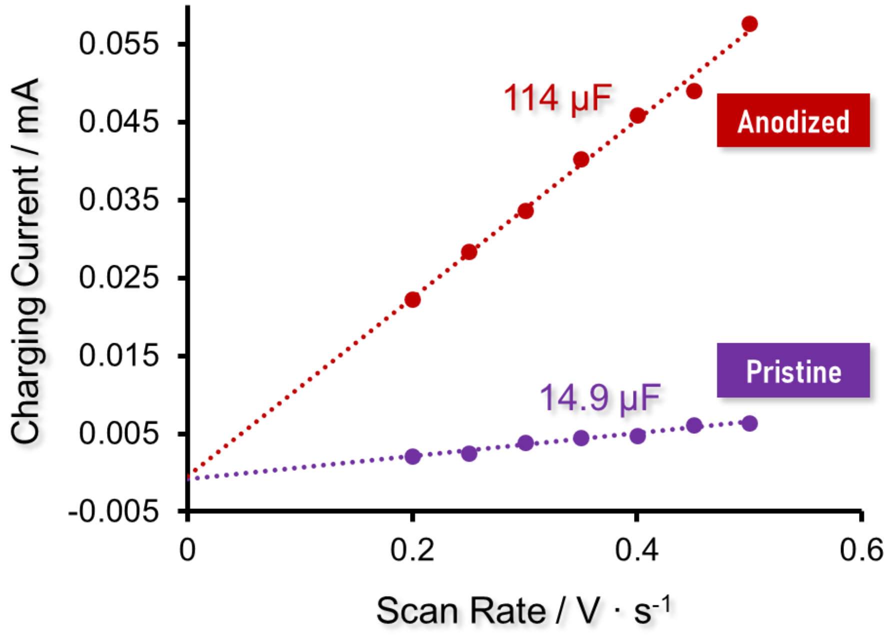

Figure S7: Electrochemical capacitance test of pristine and anodized Cu-MP. Electrolyte: CO_2-saturated 0.1 M $KHCO_3$ with CO_2 bubbling, pH = 6.8.

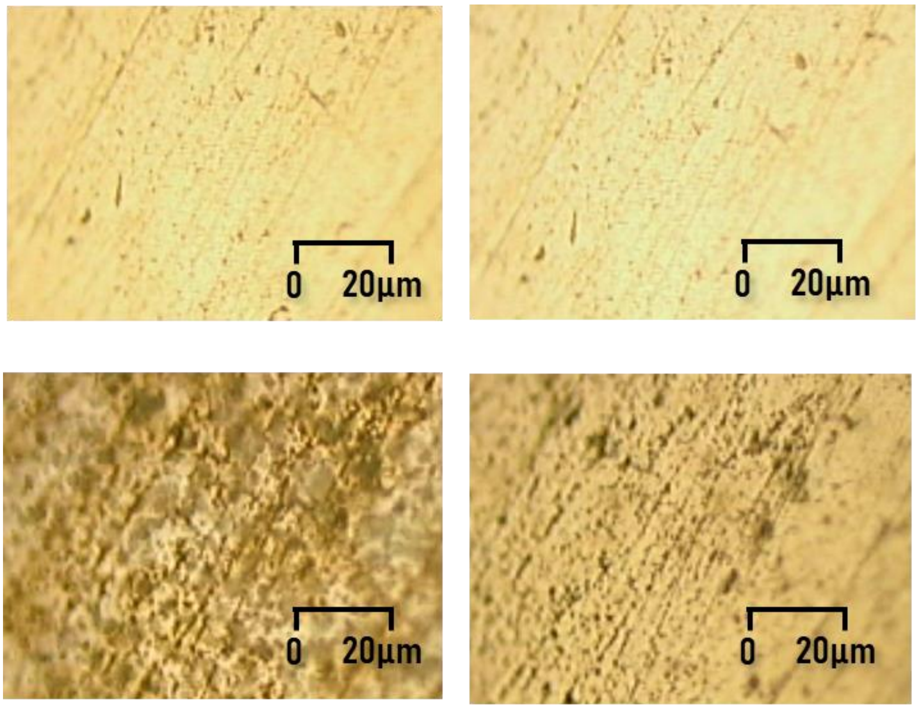

Fig. S8: Optical microscope image of Cu-MP under open-circuit potential (top left), 3 sec after -0.4 V cathodic bias (top right), 60 sec after +1.55 V anodic bias (bottom left) and 6 sec after second -0.4 V cathodic bias (bottom right). Electrolyte: CO_2-saturated 0.1 M $KHCO_3$, pH = 6.9. For video see https://youtu.be/ApNodeIgyc0

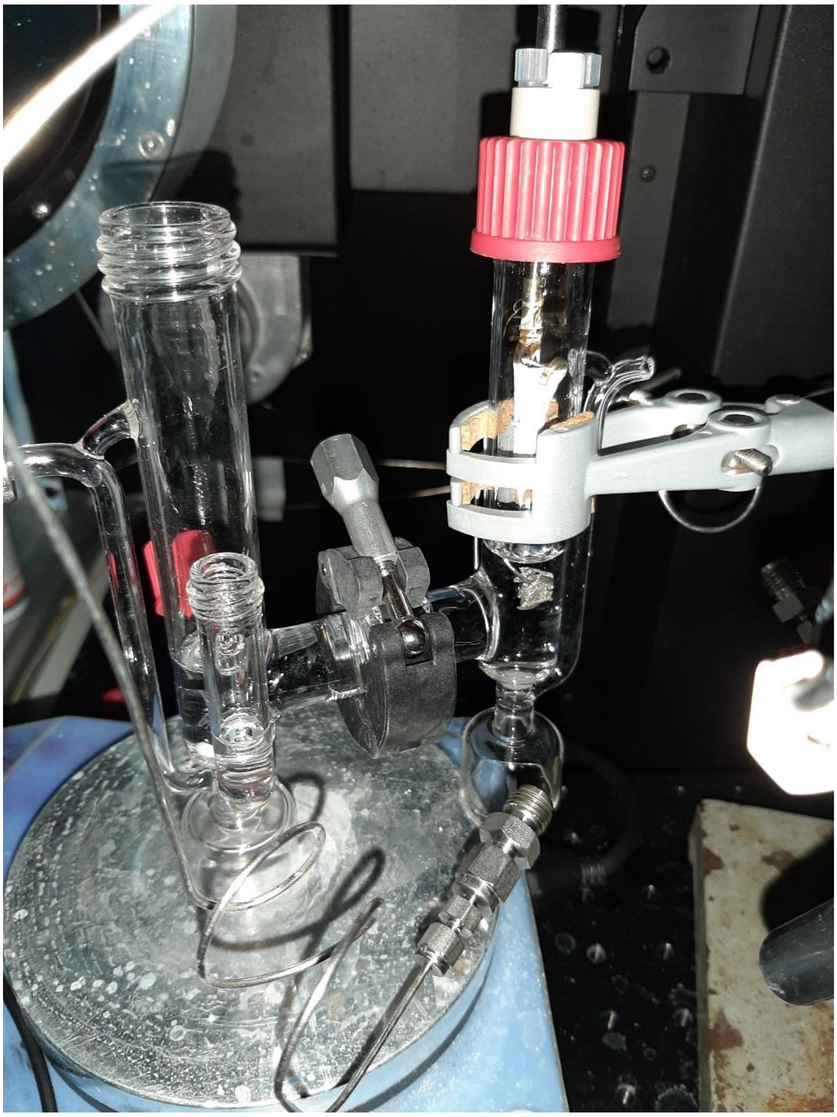

Fig. S9: H-Cell used for product analysis.

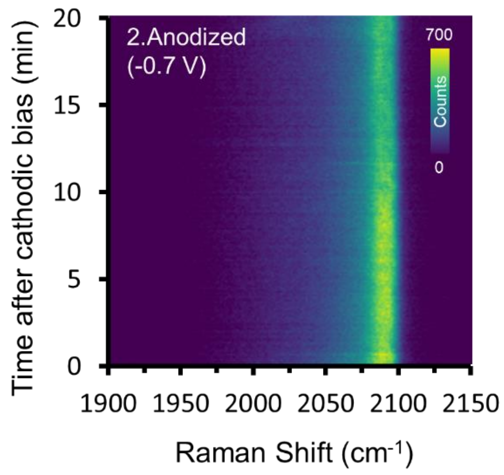

Figure S10. TR-SERS heatmap from anodized Cu-MP in the CO region (Raman shift between 1900-2150 cm^{-1}) for longer reaction times at -0.7 V vs. RHE.

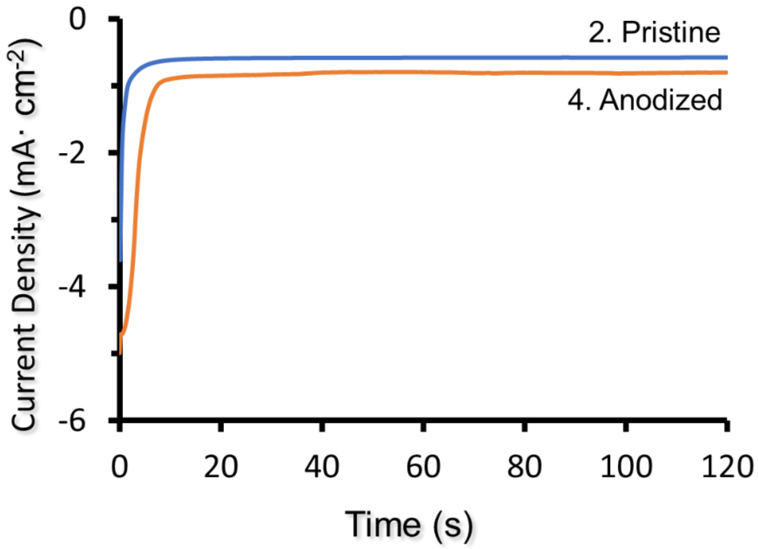

Fig. S11: Chronoamperometry (CA) curve of pristine and anodized Cu-MP during reduction at -0.7 V for 60 sec. Electrolyte: CO_2-saturated 0.1 M $KHCO_3$, pH = 6.8.

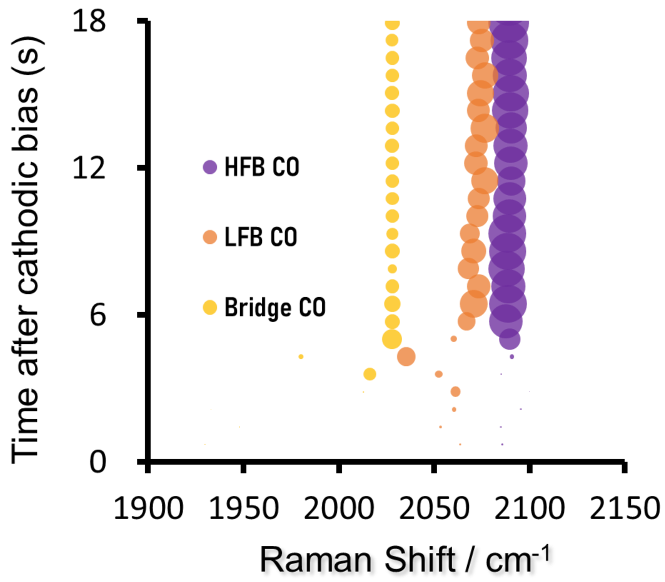

Fig. S12: Fitted result of Figure 3(b) in the main text. Bubble position shows time and Raman shift, and bubble size is proportional to peak intensity.

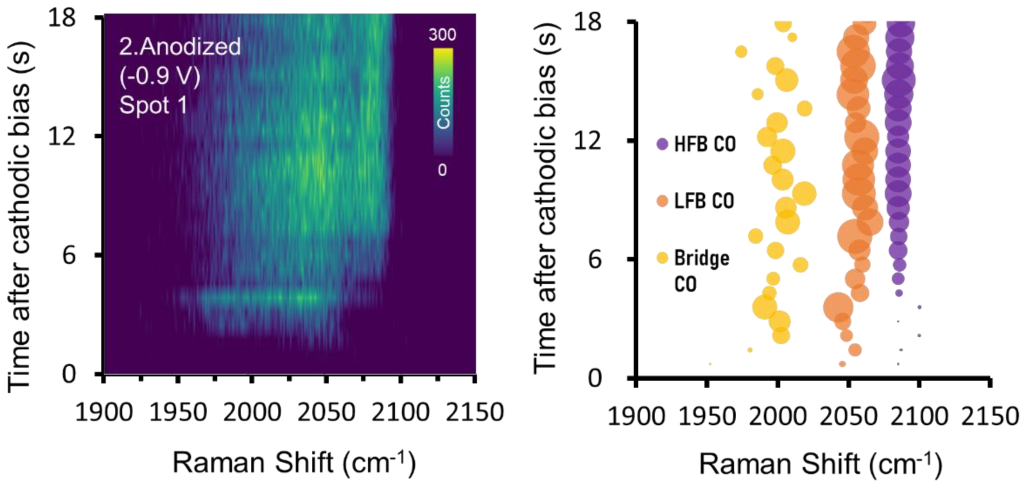

Fig. S13: Alternative observation of TR-SERS heatmap from anodized Cu-MP during reduction at -0.9 V in the CO region (Raman shift between 1900-2150 cm^{-1}). After 7 s, the HFB-CO at 2085 cm^{-1} is visible, although substantially weaker than at -0.8 V. LFB CO is stronger than at -0.8 V, and is dynamically shifting around 2050 cm^{-1}.

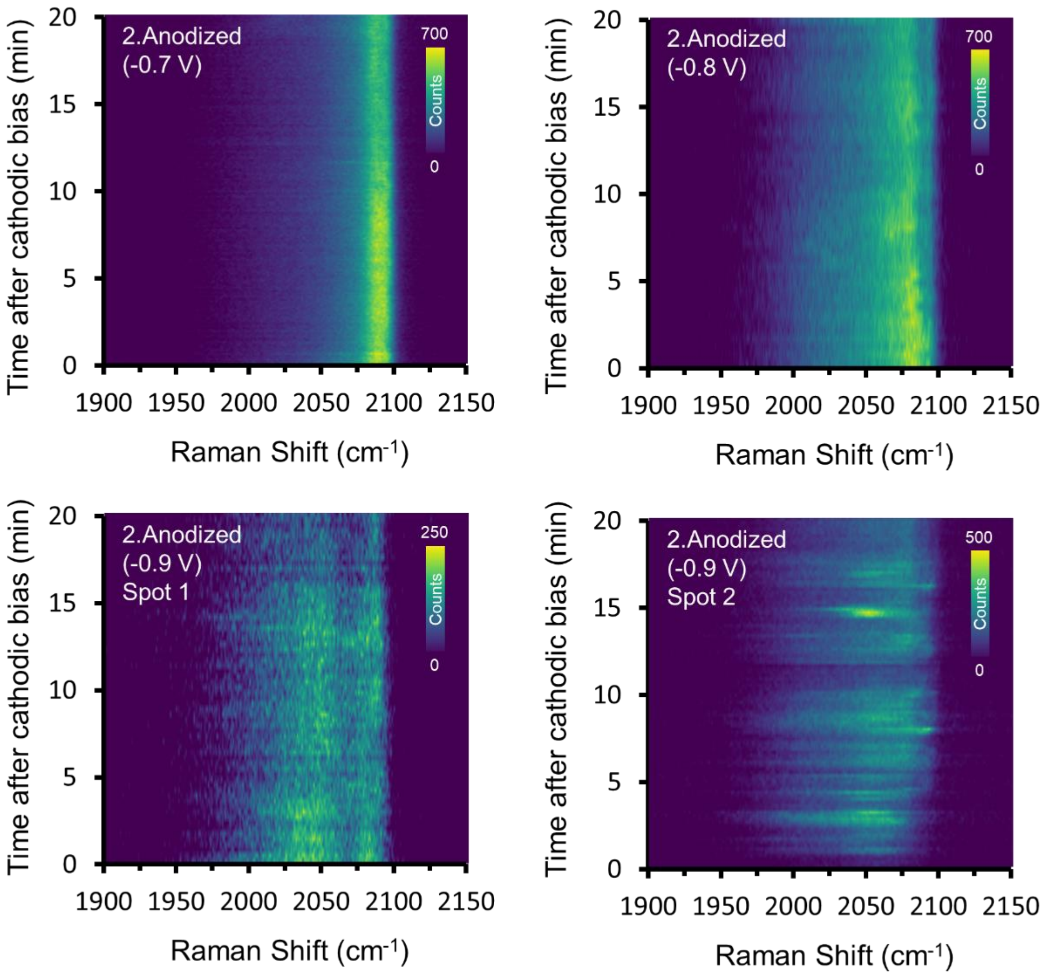

Fig. S14: TR-SERS heatmap from anodized Cu-MP in the CO region (Raman shift between 1900-2150 cm^{-1}) for longer reaction times at different cathodic bias.

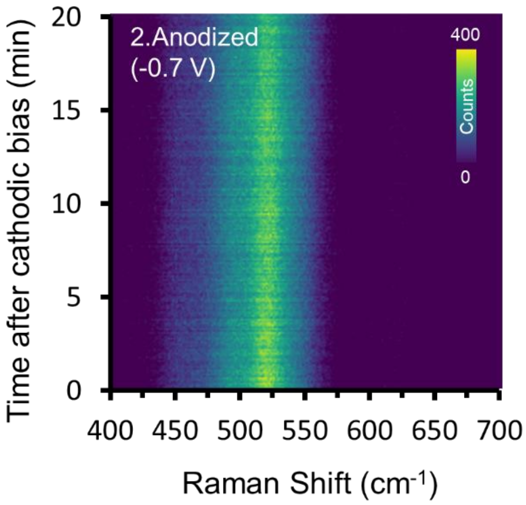

Fig. S15: TR-SERS heatmap from anodized Cu-MP in the Cu-OH region (Raman shift between 400-700 cm^{-1}) for longer reaction times.

Supporting Discussion: comparison of data with galssy carbon and Pt as counter electrode

To investigate possible Pt leaching on the electrocatalytic performance and the in situ TR-SERS data, we performed the TR-SERS experiments in an electrochemical cell with glassy carbon and Pt as counter electrode. No obvious differences between the TR-SERS data is observed, thereby excluding the possibility of Pt leaching effects on the TR-SERS data.

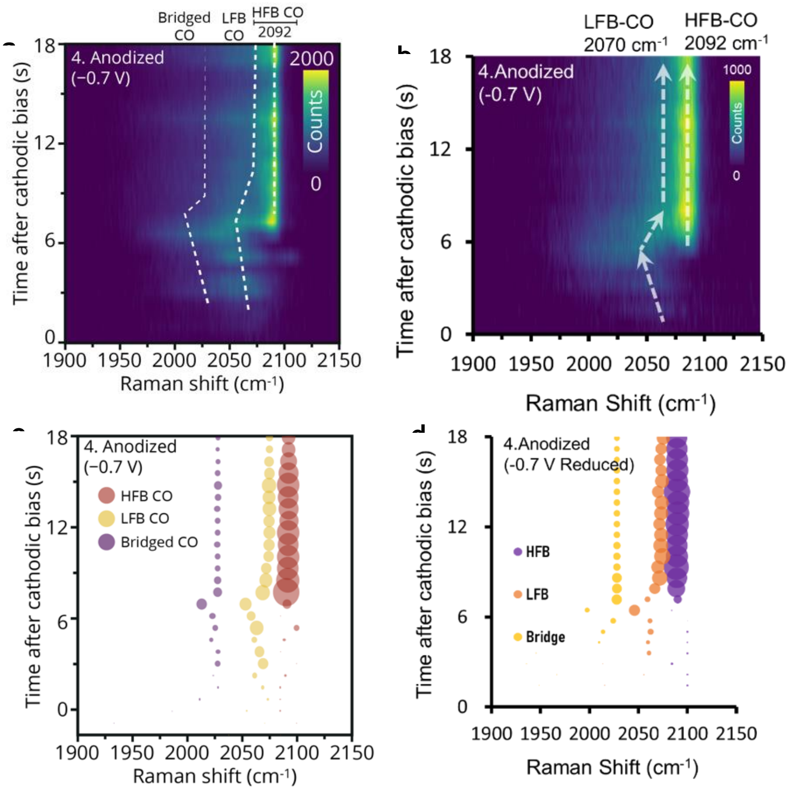

Figure S16. TR-SERS data in the CO region at -0.7 V vs. RHE with Pt (a,c) and glassy carbon (b,d) as counter electrodes.

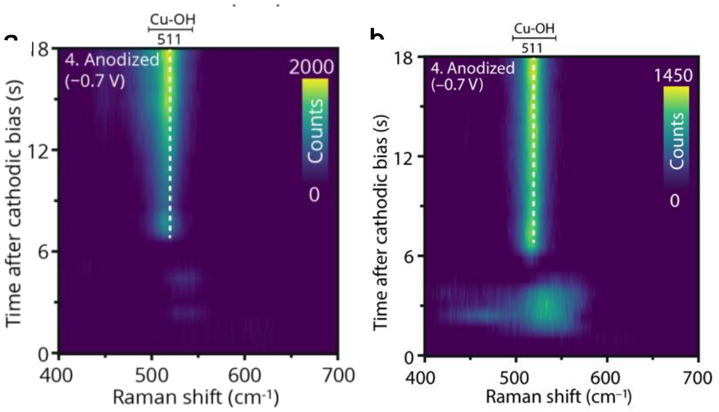

Figure S17. TR-SERS data in the oxide region at -0.7 V vs. RHE with Pt (a,c) and glassy carbon (b,d) as counter electrodes.

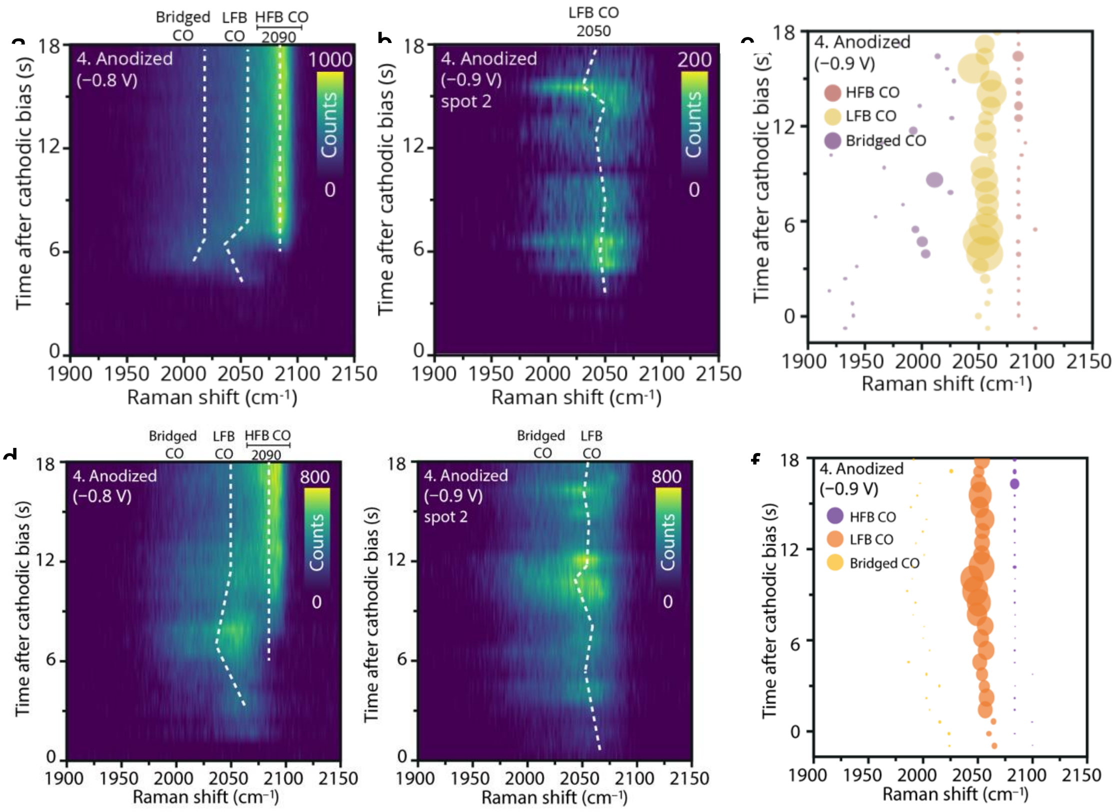

Figure S18. TR-SERS data in the CO region at -0.8 V (a,d) and -0.9 V vs. RHE (b,e) with Pt (a-c) and glassy carbon (d-f) as counter electrodes.
